# Assembly, remodelled

**DOI:** 10.7554/eLife.01270

**Published:** 2013-08-20

**Authors:** Karim Bouazoune, Robert E Kingston

**Affiliations:** 1 **Karim Bouazoune** is in the Department of Molecular Biology and the Department of Genetics, Massachusetts General Hospital, Harvard Medical School, Boston, United Statesbouazoune@molbio.mgh.harvard.edu; 2 **Robert E Kingston** is in the Department of Molecular Biology and the Department of Genetics, Massachusetts General Hospital, Harvard Medical School, Boston, United Stateskingston@molbio.mgh.harvard.edu

**Keywords:** chromatin assembly, chromatin remodelling, Chd1, nucleosome, chromatin, motor protein, *D. melanogaster*, *S. cerevisiae*

## Abstract

Biochemical assays reveal that nucleosome maturation and chromatin remodelling by the motor protein Chd1 are distinct, separable enzymatic activities.

**Related research article** Torigoe SE, Patel A, Khuong MT, Bowman GD, Kadonaga JT. 2013. ATP-dependent chromatin assembly is functionally distinct from chromatin remodeling. *eLife* **2**:e00863. doi: 10.7554/eLife.00863

**Image** Wild type (left), but not mutant (right), forms of the motor protein Chd1 can create regularly spaced arrays of nucleosomes

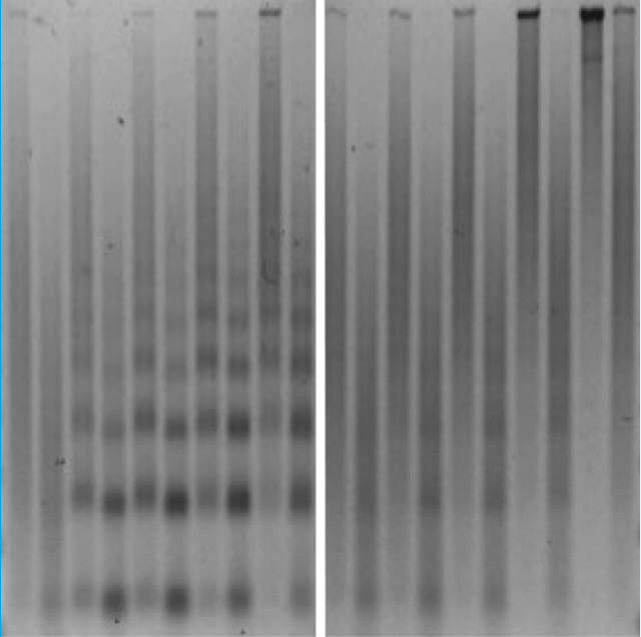

Genomic DNA must frequently be accessed in cells—most crucially, for essential activities such as the transcription of genes, the replication of chromosomes during cell division, and the repair of DNA damage. In organisms such as plants and animals, access to DNA is regulated by packaging it into a complex called chromatin, which both organizes the DNA and ensures that it is fully functional. Chromatin formation is therefore a fundamental process in biology.

Mature chromatin is an ordered array of repeating units, or nucleosomes, each comprising approximately 150 base pairs of DNA wrapped around a set of proteins known as histones. Chromatin is produced through the combined efforts of two additional types of proteins: histone chaperones passively load histones onto DNA, while motor proteins use the energy of ATP to generate nucleosome arrays. Motor proteins actually carry out two kinds of activities—completing the formation of nucleosomes, and also ensuring their regular spacing on the DNA. Now, in *eLife*, a collaboration between the laboratories of James Kadonaga, at the University of California, San Diego, and Gregory Bowman, of Johns Hopkins University, provides unexpected insight into chromatin maturation via motor proteins.

Since the 1970s, it has been clear that the histone-DNA structures initially formed by histone chaperones—which are called nascent nucleosomes—exhibit different properties from those of canonical, mature nucleosomes (Ruberti and Worcel, 1986; refs therein). For example, nascent nucleosomes are more sensitive to digestion by nucleases, enzymes that can cleave DNA. Additionally, unlike canonical nucleosomes, they do not fully retain negative supercoils (twists that result when circular DNA is wound around itself, as in a rubber band). This implies that the DNA is less tightly wrapped around the histones than in mature chromatin.

The differences between nascent and mature nucleosomes can in part be explained by how histone proteins are deposited onto DNA. Nucleosomes contain histone ‘octamers’ composed of two copies each of the core histone proteins H3, H4, H2A and H2B, which are deposited in a specific order. The prevailing model is that first, either two dimers, each with one copy of H3 and H4, or one tetramer that contains two copies each of histones H3 and H4, are loaded onto DNA. Next, two dimers, each with one copy of histones H2A and H2B, are added flanking the H3-H4 proteins (Figure 1) (for review see Macalpine and Almouzni, 2013). In vitro analyses indicate that mature chromatin is then formed by motor proteins—enzymes formally called ATP-dependent chromatin (or nucleosome) remodelling proteins. These proteins use the energy of ATP to convert the randomly deposited ‘prenucleosomes’ into mature nucleosomes (a key step in a process called ‘chromatin assembly’)*,* and then into a regularly spaced nucleosome array (utilizing a process called ‘chromatin remodelling’). This nucleosome array has the same sensitivity to digestion by nucleases as mature chromatin (Ito et al., 1997; Varga-Weisz et al., 1997; Torigoe et al., 2011; refs therein).

**Figure 1.**
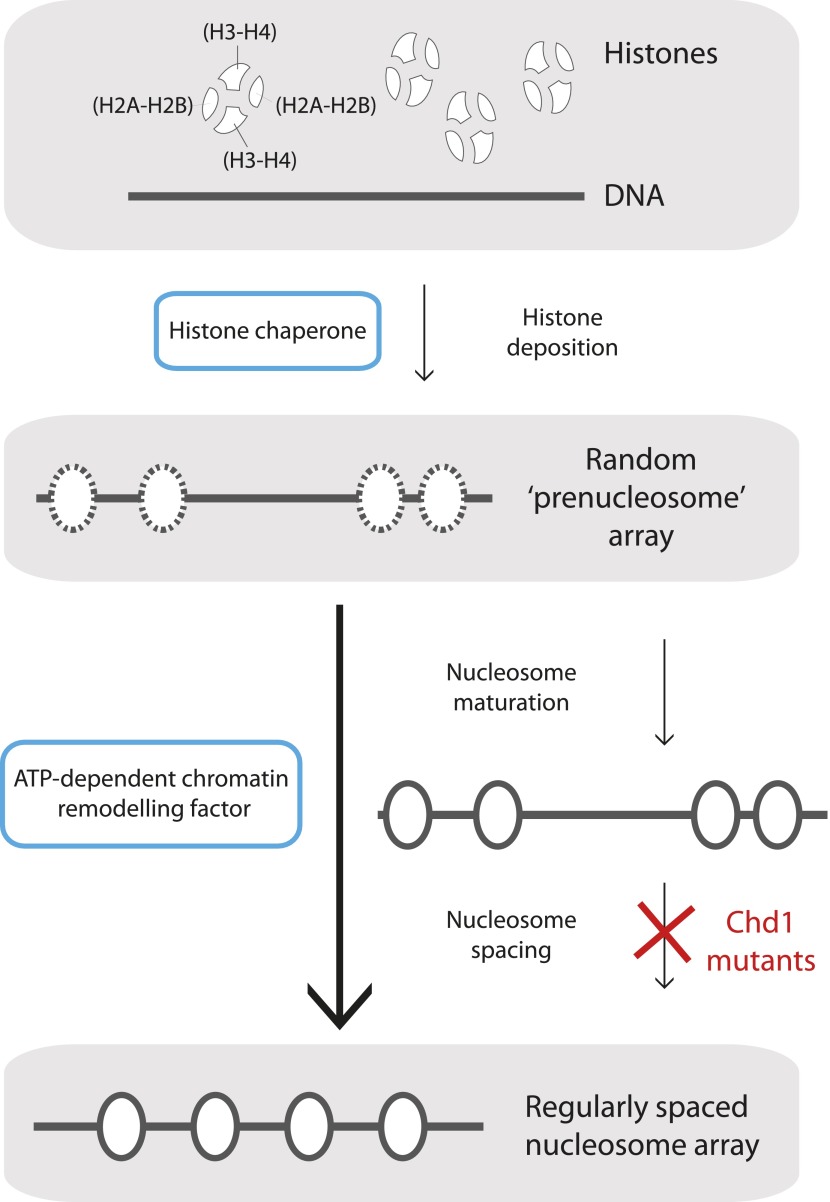
Mature nucleosome arrays are formed in several stages. Histone proteins are first loaded onto DNA to create non-canonical nucleosomal structures referred to as ‘prenucleosomes’ (top and second panels). The core histones, H2A, H2B, H3 and H4, associate with each other in heterodimers (H2A-H2B and H3-H4; top panel) and are deposited onto DNA in a specific order by proteins called histone chaperones (top blue box) to form an irregularly spaced prenucleosome array (dashed ovals, second panel). Mature nucleosomes are then formed, and also repositioned to ensure their even spacing on the DNA, by ATP-dependent chromatin remodelling factors (motor proteins; lower blue box). Maturation and repositioning were formerly thought to be achieved by a single enzymatic function (long arrow at left, bottom panel). Now, using mutant variants of a motor protein called Chd1, Torigoe et al. suggest that chromatin remodelling factors use not one but two distinct ATP-dependent activities to convert randomly deposited prenucleosomes into a mature array of evenly spaced nucleosomes (solid ovals; short arrows at right, third and bottom panels).

Chromatin, or nucleosome, remodelling is shorthand for a set of related processes that can be carried out on mature nucleosomes, and that can occur either as chromatin is formed or during cellular events such as gene transcription. These include ‘sliding’ (repositioning) of the histone octamer along DNA; the formation of regularly spaced, or periodic, nucleosome arrays; the deposition or eviction of histone octamers; and the exchange of core histone proteins for histone variants (for review Hopfner et al., 2012; Clapier and Cairns, 2009). Most of the ATP-dependent chromatin remodelling proteins that perform these processes belong to four major related families: SWI/SNF (SWItch/Sucrose-Non-Fermenting); ISWI (Imitation SWItch); CHD (Chromodomain Helicase DNA-binding); and SWR1/INO80 (Swi2/Snf2-Related 1/ Inositol Requiring 80). These enzymes use a region called the ATPase/DNA-translocase domain to remodel chromatin; studies have established that the different subfamilies catalyse different reactions.

The Kadonaga and Bowman groups—including Sharon Torigoe as first author—joined forces to dissect the relationship between the maturation of prenucleosomes into nucleosomes and the remodelling of fully formed nucleosomes. They studied a CHD family protein, Chd1, and took advantage of mutant versions of yeast Chd1 that retain significant ATPase activity, but cannot remodel chromatin. Specifically, these mutants are extremely inefficient at sliding nucleosomes along a DNA fragment (Patel et al., 2011). As expected, these chromatin-remodelling–defective Chd1 proteins were unable to space nucleosomes periodically. Surprisingly, however, they could still support nucleosome assembly from prenucleosomes in the presence of ATP. This provides evidence, for the first time, that the maturation and remodelling of nucleosomes are distinct processes.

Kadonaga and co-workers found further evidence that these processes were separable by investigating a protein called BRG1, which functions as the ATPase subunit of the human SWI/SNF complex. BRG1 remodels chromatin, but the authors observed that it could not catalyse the maturation of prenucleosomes, indicating that ATP-dependent chromatin remodelling is not sufficient to convert prenucleosomes into nucleosomes (i.e., to complete nucleosome assembly). Thus, at least in vitro, the formation of regularly spaced nucleosome arrays seems to require two distinct ATP-dependent activities.

These findings cumulatively led Torigoe et al. to suggest a model for chromatin formation in which histone chaperones first deposit histones onto DNA to create prenucleosomes (Torigoe et al., 2011). Next, specific chromatin remodelling factors use the energy of ATP to convert these intermediates into canonical nucleosomes, which are then (simultaneously or subsequently) remodelled into periodic arrays (Figure 1).

Many chromatin assembly studies have shown that nascent nucleosomes are more sensitive to nuclease digestion than mature nucleosomes, and that the maturation of chromatin depends on the energy of ATP (Ruberti and Worcel, 1986; refs therein). While some of these initial structures have been attributed to the sequential loading of histone dimers or tetramers onto DNA, Kadonaga and co-workers propose a new fundamental step in the process of chromatin maturation. It is now essential to determine how these biochemical observations relate to the chromatin assembly pathways undertaken during DNA replication, DNA repair and gene transcription in vivo (Macalpine and Almouzni, 2013). Does the formation of chromatin in vitro reflect events in vivo fully, or only incompletely? In particular, organisms have many histone chaperones; some, such as CAF-1 (Chromatin Assembly Factor 1), have different properties than NAP1 (Nucleosome Assembly Protein 1), the chaperone used by Torigoe et al. These differences might influence the way in which chromatin matures. Nonetheless, by identifying Chd1 mutants that can still catalyse nucleosome maturation despite defects in chromatin remodelling, the current study provides a new angle to explore these issues in vivo.
